# Damped resonance for broadband acoustic absorption in one-port and two-port systems

**DOI:** 10.1038/s41598-019-49222-w

**Published:** 2019-09-10

**Authors:** Taehwa Lee, Tsuyoshi Nomura, Hideo Iizuka

**Affiliations:** Toyota Research Institute of North America, Toyota Motor North America, Ann Arbor, Michigan 48105 USA

**Keywords:** Mechanical engineering, Applied physics, Acoustics

## Abstract

We demonstrate broadband perfect acoustic absorption by damped resonances through inclusion of lossy porous media. By minimally placing the lossy materials around the necks of single-resonance Helmholtz resonators, where acoustic energy is concentrated, we show an increase in absorption bandwidths (>100% of the resonance frequency). Using the damped resonance, we demonstrate three types of broadband acoustic absorbers in one-port and two-port systems: broadband absorbers (one-port), broadband sparse absorbers (two-port), and broadband duct absorbers (two-port). Our approach for broadband absorption allows to minimize the number of resonances for compact absorbers, while it is beneficial for practical applications owing to the minimum use of porous materials.

## Introduction

Acoustic metamaterials allow unprecedented control of sound waves^1–4^, enabling intriguing phenomena such as acoustic cloak^5^, asymmetrical transmission^6,7^, topological insulators^8,9^, and perfect absorbers^10–13^. Especially, acoustic metamaterials for perfect absorption lead to lots of practical applications for noise and vibration control, since they have advantages over conventional acoustic absorbers, e.g., for low-frequency sound absorption^10,11^. Despite such an advantage, resonance-based acoustic metamaterials often suffer from a relatively narrow absorption bandwidth. There have been extensive efforts devoted to increasing absorption bandwidths^12,13^. The most common approach uses multiple resonances, whose frequencies are closely placed for overlap of their absorption spectra^12–14^. Recently, multi-resonance metamaterials overlaid with a thin lossy medium have shown an extremely broadband acoustic absorption^14^.

Some complexities are involved in designing such multi-resonance metamaterials. For example, each resonance in such a metamaterial should fulfill critical coupling conditions^12–14^. In addition, combination of multiple resonators each having a different resonance frequency leads to a bulky structure. Thus, minimizing the number of resonators in a unit cell is practically desired for constructing compact broadband absorbers. One approach implements multi-order resonances of one resonator in the unit cell, simultaneously satisfying critical coupling conditions at these multi-order resonances^15^. In this device, the spacing between the individual resonances is restricted by the fundamental frequency and its higher harmonics. Alternatively, ultra-broadband absorption can be realized with a single-resonance resonator in the unit cell, which has been theoretically demonstrated^16^. In addition, single-resonance broadband absorbers operating in water have been reported^17^. For airborne absorbers, broadband absorption has been demonstrated by combining a lossy medium with acoustic resonances^18–24^, proving the effectiveness of damped resonance.

In this work, we demonstrate broadband airborne acoustic absorption by maximizing the contribution of damped resonance. We first discuss conditions for perfect absorption in systems with and without transmission channels. For damped resonance, a lossy porous medium is minimally implemented in the regions of Helmholtz resonators where acoustic pressure field is highly concentrated. The effect of the lossy material on acoustic performance is systematically investigated. We demonstrate three types of broadband absorbers based on damped resonance.

## Results

### Perfect acoustic absorption in one-port and two-port systems

**Figure 1:**
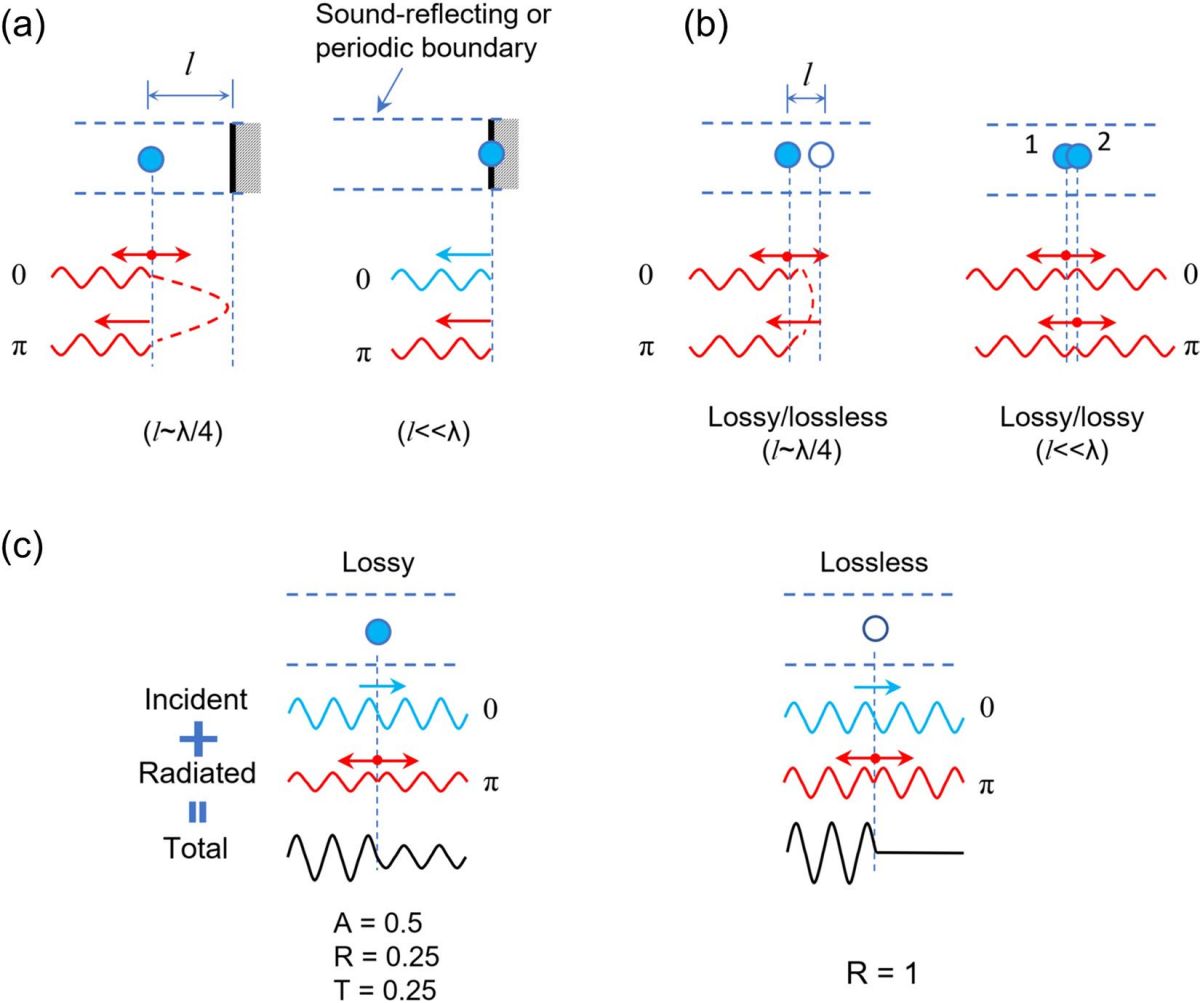
Perfect acoustic absorbers in one-port and two-port systems. (**a**) Single-resonance resonators in one-port systems. For a resonator away from a reflector ($l\ne 0$), a lossy resonator has perfect absorption when the backward-radiated waves from the resonator are cancelled by the reflected waves of the forward-radiated waves by the reflector, thus requiring a relative phase shift of π and a quarter-wavelength distance $l\sim \lambda /4$. For a resonator on the reflector (*l* = 0), perfect absorption occurs when the reflected waves from the reflector is directly cancelled by the radiated waves from the resonator. (**b**) Dual-resonance resonators in two-port systems. For lossy/lossless resonators, perfect absorption occurs when $l\sim \lambda /4$, as the lossless resonator functions as a reflector. For lossy/lossy resonators, de-tuning of their resonance frequencies is needed to cancel the radiated waves out of each resonator. The distance between the resonators can be much smaller than the wavelength ($l\ll \lambda$). (**c**) Single-resonance resonators in two-port systems. A lossy single-resonance resonator has 0.5 absorption, 0.25 reflection, and 0.25 transmission, whereas a lossless resonator functions as a reflector with no transmission (i.e. the forward-radiated waves from the resonator are out-of-phase with the incident waves).

As the acoustic absorption (*A*) is given by $A=1-|r{|}^{2}-|t{|}^{2}$ with the reflection coefficient *r* and the transmission coefficient *t*, perfect acoustic absorption (*A* = 1) is ensured when reflection and transmission are zero (i.e. $R=|r{|}^{2}=0$ and $T=|t{|}^{2}=0$). The perfect absorption requires different approaches, depending on presence of transmission channels. For one-port systems, where transmission is prohibited by reflectors backing resonators (i.e. *T* = 0), the perfect acoustic absorption condition is equivalent to a zero-reflection condition (*R* = 0), requiring the impedance matching^11,25^. As illustrated in Fig. 1(a), one-port systems are composed of acoustic resonators and an acoustic reflector, and the distance (*l*) between the resonator and the reflector can be chosen as either $l\ll \lambda$ or $l\sim \lambda /4$. For two-port systems, where acoustic transmission channels exist, besides zero reflection, transmission needs to be zero in order to demonstrate perfect acoustic absorption. Transmission in two-port systems results from the radiation symmetry. For zero transmission, breaking the symmetry of systems has been implemented for a relatively small open channel^13^. For systems with relatively large open channels, resonance degeneracy (or dual resonance) is required to demonstrate perfect absorption^26–28^. As seen in Fig. 1(b), for perfect absorption, one can use two approaches based on dual resonances consisting of lossy/lossy^28^ or lossy/lossless resonators^29^.

To understand the need of such resonance degeneracy, consider a single resonance in a two-port system, as illustrated in Fig. 1(c). For a lossy subwavelength resonator, the theoretical absorption limit is *A* = 0.5 because of the radiation symmetry (*R* = 0.25 and *T* = 0.25)^30^. In this case, perfect absorption is possible only when two acoustic waves are coherently incident from the opposite directions (i.e. coherent perfect absorption mechanism)^31,32^. In contrast, a lossless subwavelength resonator in a two-port system works as a perfect reflector^33^, because the radiated wave from the resonator is out-of-phase with the incident wave (i.e. destructive interference).

From an interference perspective, perfect acoustic absorption is explained for one-port and two-port systems, as shown in Fig. 1(a,b). For zero reflection in one-port systems, the backward radiated wave of a single-resonance resonator should be cancelled by reflected waves from the reflector that is placed with a quarter-wavelength distance ($l=\lambda /4$). When a single-resonance resonator is placed on a reflector, the reflected wave from the reflector should be directly cancelled with the radiated wave from the resonator^30^. In this case, the area ratio between the resonator and the surrounding reflector is critical. On the other hand, for two-port systems, the perfect absorption of lossy/lossless resonators is realized by the lossless subwavelength resonator that functions as a reflector (i.e. no transmission). When dual resonances result from lossy/lossy resonators, they need to be slightly de-tuned for perfect absorption^28^. Such de-tuning leads to a π phase difference between the radiated waves from the two resonators, resulting in cancellation of these radiated waves. These de-tuned resonators can be placed in close proximity at a distance of $l\ll \lambda$.

### Theoretical absorption bandwidth limit

For single-resonance resonators in one-port systems, the peak absorption (*A*) is expressed by

1 $$A=\frac{4{C}_{leak}{C}_{loss}}{{({C}_{leak}+{C}_{loss})}^{2}},$$

where *C*_*leak*_ is the radiation leakage (kg/s), and *C*_*loss*_ is the intrinsic loss (kg/s). The detailed derivation of Eq. (1) can be found in Methods. From Eq. (1), the maximum absorption occurs when ${C}_{leak}={C}_{loss}$, i.e. the critical coupling condition stating that the radiation leakage (*C*_*leak*_) out of the resonator should be matched with the intrinsic losses (*C*_*loss*_)^18,34^. In general, the leakage rate has a complex value^35^. Under the assumption ($d\ll \lambda$), the leakage rate can be expressed as a simple form. For two-dimensional (2D) resonators on a reflector with a period of $d\ll \lambda$, the radiation leakage (kg/s per unit depth) is given by^17^

2 $${C}_{leak}={Z}_{s}\frac{{s}^{2}}{d},$$

where *s* is the width of the vibrating mass, *d* is the period and *Z*_*s*_ is the acoustic impedance of the surrounding fluid (${Z}_{s}={\rho }_{s}{c}_{s}$; ${\rho }_{s}$ density, *c*_*s*_ sound speed) (see Supplementary information). When resonators are isolated in free field^36^, or the period is comparable to the wavelength, the acoustic reactance (i.e. imaginary part of *C*_*leak*_) is non-negligible^37^. Note that *C*_*leak*_ is proportional to *s*^2^ and inversely proportional to *d*, indicating that the critical coupling (*C*_*leak*_ = *C*_*loss*_) can be realized by adjusting these geometrical parameters. In addition, *C*_*leak*_ is proportional to *Z*_*s*_; the radiation leakage from resonators in water is much higher than that from the same resonators in air, i.e. ${C}_{leak,{\rm{water}}} > {C}_{leak,{\rm{air}}}$.

By using the fact that the quality factor (${f}_{res}/\Delta {f}_{0.5}$) is inversely proportional to damping ratio $(\xi =\frac{{C}_{leak}+{C}_{loss}}{{C}_{c}})$^38^, the full-width-at-half-maximum (FWHM) absorption bandwidth (Δ*f*_0.5_) is given by

3a $$\begin{array}{rcl}\Delta {f}_{0.5} & = & 2\frac{{C}_{leak}+{C}_{loss}}{{C}_{c}}{f}_{res},\end{array}$$

3b $$\begin{array}{rcl} & = & \frac{1}{2\pi }\frac{{C}_{leak}+{C}_{loss}}{{m}_{0}},\end{array}$$

where *C*_*c*_ is the critical damping, *f*_*res*_ is the resonance frequency (${C}_{c}/{f}_{res}=4\pi {m}_{0}$), and *m*_0_ is the vibrating mass of a resonator (${m}_{0}={\rho }_{v}sh$ per unit depth with ${\rho }_{v}$ the density, *s* the width, and *h* the height). For the critical coupling (${C}_{leak}={C}_{loss}$), Eq. (3b) becomes $\Delta {f}_{0.5}=\frac{1}{\pi }\frac{{C}_{leak}}{{m}_{0}}=\frac{1}{\pi }\frac{{\rho }_{s}}{{\rho }_{v}}\frac{{c}_{s}s}{dh}$, depending on ${\rho }_{s}/{\rho }_{v}$.

From Eq. (3b), we can identify the fundamental difference in absorption bandwidth between membrane-type resonators and Helmholtz resonators. Membrane-type resonators operating in air have a relatively narrow bandwidth because of a large density difference $\frac{{\rho }_{s}}{{\rho }_{v}}\sim {10}^{-3}$, thus requiring a thin membrane (i.e. small *h*) to compensate such a difference. However, when operating in water, membrane-type resonators enable a much broader bandwidth owing to the similar mass density $(\frac{{\rho }_{s}}{{\rho }_{v}}\sim 1)$^17^. On the other hand, Helmholtz resonators are effective for broadband absorption in both air and water, because their vibrating masses in the necks have the same density as the surrounding fluid, i.e. $\frac{{\rho }_{s}}{{\rho }_{v}}=1$. Thus, the absorption bandwidth of Helmholtz resonators (neck width *w*_*n*_, neck length *l*_*n*_) is given by $\Delta {f}_{0.5}=\frac{1}{\pi }\frac{{c}_{s}}{d}\frac{{w}_{n}}{({l}_{n}+\varepsilon {w}_{n})}$ with $s={w}_{n}$ and $h={l}_{n}+\varepsilon {w}_{n}$ (end correction factor $\varepsilon$). The absorption bandwidth can be maximized to ~$\frac{{c}_{s}}{d\varepsilon }$ when $\frac{{w}_{n}}{{l}_{n}}\gg 1$, indicating that broadband absorption is enabled by small *d* and large $\frac{{w}_{n}}{{l}_{n}}$ (i.e. wide neck width and short neck length).

### Bandwidth of perfect acoustic absorbers

**Figure 2:**
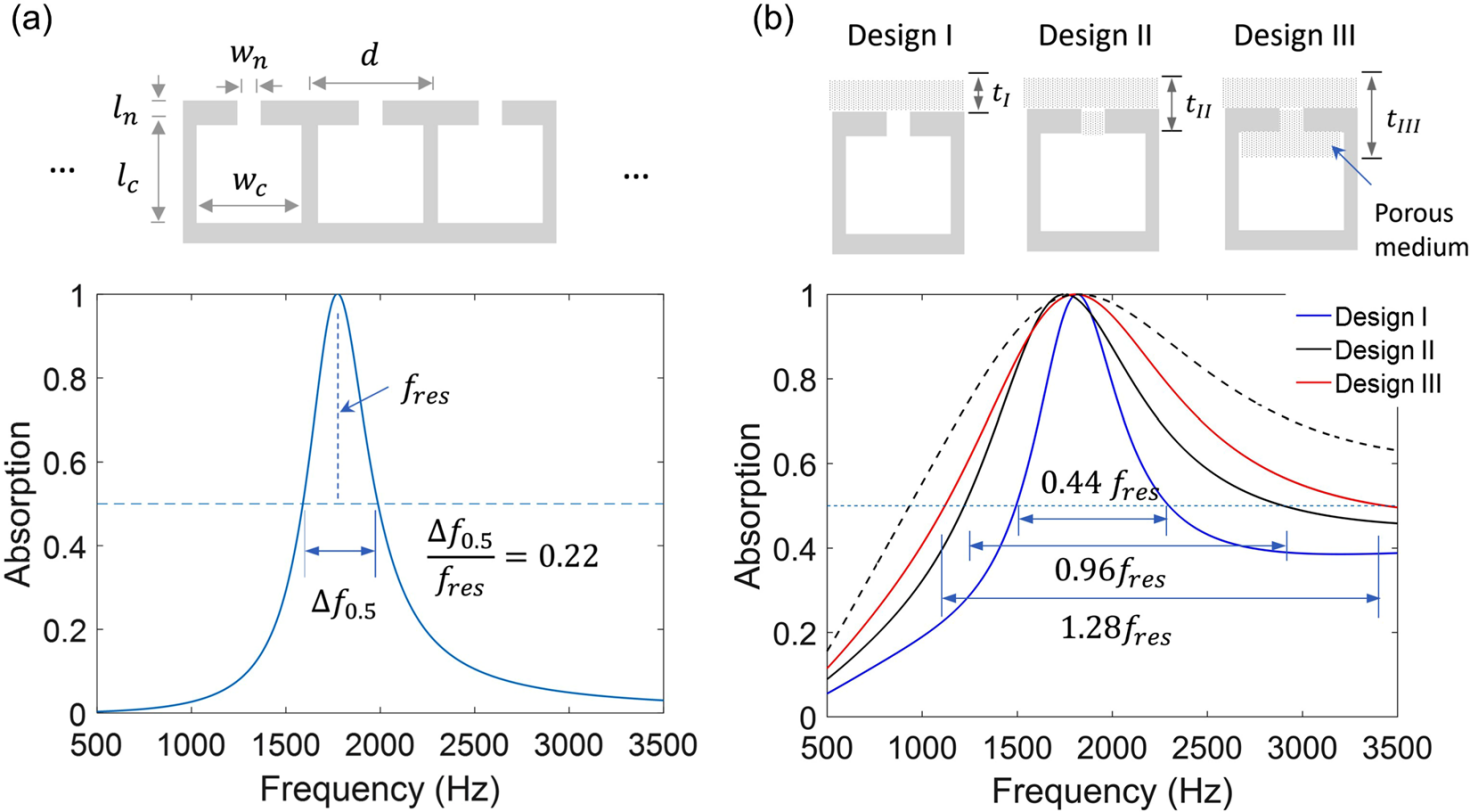
Absorption bandwidth of Helmholtz resonators. (**a**) Absorption spectrum and bandwidth of Helmholtz resonators relying on neck friction losses. The dimensions of the resonators are given by the period (*d* = 25 mm), the neck size (*w*_*n*_ = 0.5 mm, *l*_*n*_ = 4 mm), and the cavity size (*w*_*c*_ = 5.5 mm, *l*_*c*_ = 15 mm). The resonator satisfies the critical coupling condition, i.e. *C*_*leak*_ = *C*_*loss*_ = 0.0041 kg/s m (or *C*_*loss*_/*m*_0_ = 1193 s^−1^). (**b**) Helmholtz resonators with inclusions of lossy media into different regions: Design I (*w*_*n*_, *l*_*c*_) = (0.5 mm, 12 mm); Design II (*w*_*n*_, *l*_*c*_) = (2 mm, 28 mm); Design III (*w*_*n*_, *l*_*c*_) = (4 mm, 34 mm). The other dimensions remain constant (*d* = 28 mm, *l*_*n*_ = 2 mm, and *w*_*c*_ = 11 mm). All the configurations fulfil the critical coupling condition, i.e. for Design I, II, and III, *C*_*leak*_ = *C*_*loss*_ = 0.0037, 0.0588, and 0.2352 kg/s m (or *C*_*loss*_/*m*_0_ = 1801, 3951, and 4711 s^−1^), respectively. The absorption spectrum and bandwidth of each configuration are compared. The black dashed line represents the absorption spectrum of resonators having cavities [(*w*_*n*_, *l*_*c*_) = (2 mm, 28 mm)] completely filled with foams. Each foam layer has a thickness of *t*_*I*_ = 10 mm, *t*_*II*_ = 12 mm, or *t*_*III*_ = 22 mm for Design I, II, or III.

To analyze the absorption bandwidth, we consider single-resonance Helmholtz resonators in a one-port system (Fig. 1(a)). Figure 2(a) shows the numerically-calculated absorption spectrum of Helmholtz resonators with the period (*d* = 25 mm), the neck size (*w*_*n*_ = 0.5 mm, *l*_*n*_ = 4 mm), and the cavity size (*w*_*c*_ = 5.5 mm, *l*_*c*_ = 15 mm). The absorber demonstrates the perfect absorption with a bandwidth of Δ*f*_0.5_ = 395 Hz (0.22*f*_*res*_), satisfying the critical coupling condition (*C*_*loss*_ = *C*_*leak*_ = 0.0041 kg/s m). By using Eqs (2) and (3), the absorption bandwidth is estimated to be $\Delta {f}_{0.5}=\frac{{Z}_{s}{w}_{n}^{2}}{\pi d{m}_{0}}=379\,{\rm{Hz}}$ (0.21*f*_*res*_) with the vibrating air mass within the neck (${m}_{0}={\rho }_{{\rm{air}}}{w}_{n}{l}_{n}^{eff}$) and the effective neck length [${l}_{n}^{eff}={w}_{n}/{w}_{c}{l}_{c}{(2\pi {f}_{res})}^{2}$ or ${l}_{n}^{eff}={l}_{n}+\varepsilon {w}_{n}$ with end correction]. A critical question arises whether the absorption bandwidth can be further increased by changing the physical dimensions (e.g. *w*_*n*_, *l*_*n*_, or *d*) without introducing a more lossy material. We find that the absorption bandwidth of Helmholtz resonators (Δ*f*_0.5_/*f*_*res*_) is fundamentally limited to ~0.2 for the perfect absorption (*A* = 1) regardless of the physical dimensions.

To understand the fundamental bandwidth limit, one can consider increasing *C*_*loss*_ by increasing the neck length (*l*_*n*_). This approach is contradictory to the derived condition of $\frac{{w}_{n}}{{l}_{n}}\gg 1$. Moreover, increasing *l*_*n*_ leads to an increase in the vibrating mass (${m}_{0}\propto {l}_{n}$) and, therefore, both $\frac{{C}_{loss}}{{m}_{0}}$ (=*c*^0^ $\cong$ 1200 s^−1^) and the bandwidth Δ*f*_0.5_ remain constant (see Eq. (3b)). Thus, the fundamental bandwidth limit is set with the maximum of $\frac{{C}_{loss}}{{m}_{0}}$, which is rather insensitive to the physical dimensions. In this regard, porous media are effective in increasing the intrinsic losses (*C*_*loss*_) without increasing the vibrating mass (*m*_0_) due to their high porosity. Evidently, lossy porous materials (e.g. foams) have been used in combination of resonators, increasing the absorption bandwidth^13,19,20^.

To maximize the effect of lossy media on the absorption bandwidth, we investigate Helmholtz resonators with different inclusions of lossy media (Design I, II, and III), as illustrated in Fig. 2(b). Figure 2(b) also shows the absorption spectra of the three configurations. The neck width (*w*_*n*_) and the cavity length (*l*_*c*_) of the resonators are adjusted to ensure ${C}_{loss}={C}_{leak}$ and the same resonance frequency, while the other dimensions remain constant (*d* = 18 mm, *l*_*n*_ = 2 mm, and *w*_*c*_ = 11 mm). With increasing *C*_*loss*_ by the lossy foams, *C*_*leak*_ should be increased by using a larger neck width (*w*_*n*_) or smaller period (*d*) (see Eq. (2)). By covering with lossy foams (Design I; *w*_*n*_ = 0.5 mm, *l*_*c*_ = 12 mm), the absorption bandwidth is increased by a factor of two ($\Delta {f}_{0.5}=0.44{f}_{res}$), compared to the resonator without a lossy medium (Fig. 2(a)). Such an increase results from both increased leakage and intrinsic losses (*C*_*loss*_ = *C*_*leak*_ = 0.0037 kg/s m; *C*_*loss*_/*m*_0_ = 1801 s^−1^ = 1.49*c*^0^). Notably, filling the neck with lossy foams (Design II; *w*_*n*_ = 2 mm, *l*_*c*_ = 28 mm) significantly increases the bandwidth to $\Delta {f}_{0.5}=0.96{f}_{res}$ due to *C*_*loss*_/*m*_0_ = 3.29*c*^0^. By partially filling the cavity (Design III; *w*_*n*_ = 4 mm, *l*_*c*_ = 34 mm), the bandwidth is further increased to $\Delta {f}_{0.5}=1.28{f}_{res}$ (a slight increase in *C*_*loss*_/*m*_0_ = 3.93*c*^0^). For comparison, the spectrum of a resonator (*w*_*n*_ = 4 mm, *l*_*c*_ = 34 mm) having the cavity completely filled with the foam is plotted (dashed line), showing only a slight increase in bandwidth. Intuitively, placing lossy media near a region, where the acoustic energy is concentrated, would be effective in maximizing the acoustic loss while minimizing the use of lossy media.

**Figure 3:**
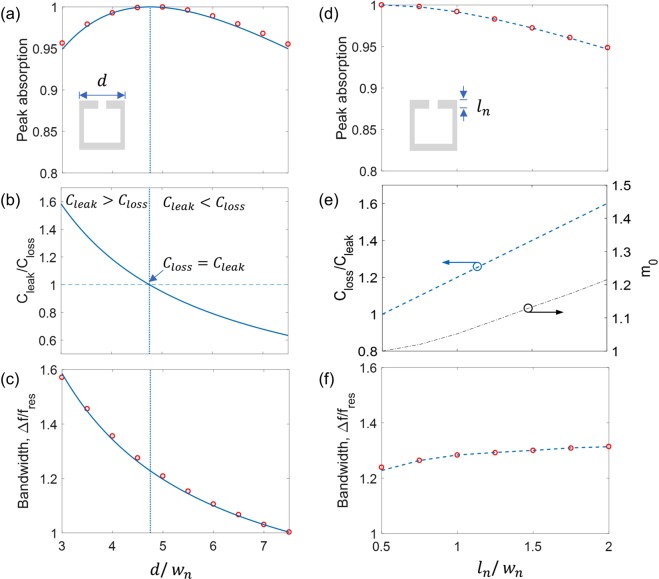
Limits of absorption bandwidth. (**a**) Peak absorption with respect to the period *d*. The symbols indicate numerical results, while the solid line is analytically obtained from Eq. (1). (**b**) Radiation leakage rate (*C*_*leak*_) for different *d* and constant intrinsic loss (*C*_*loss*_). *C*_*leak*_ is calculated by Eq. (2). (**c**) Dimensionless absorption bandwidth for different *d*. The numerical results (symbols) are compared with the analytical result (solid line) using Eq. (3). (**d**) Peak absorption with respect to the neck length *l*_*n*_. The dashed line represents the fit line using Eq. (1) with assumed linear intrinsic losses (*C*_*loss*_). (**e**) Assumed intrinsic loss (blue dashed line) and vibration mass (black dash-dotted line) used for the fit lines of the peak absorption and absorption bandwidth. (**f**) Dimensionless absorption bandwidth for different *l*_*n*_.

For Design III, we further study the effects of the period (*d*) and the neck length (*l*_*n*_) on the absorption bandwidth. With varying only *d*, the leakage rate (*C*_*leak*_) is changed while the loss (*C*_*loss*_) remains the constant. There is no change in the cavity size and thus constant resonance frequency. Figure 3(a) shows peak absorption for different *d*. The numerical results (symbols) show the good agreement with the analytical results (solid line), which are calculated from Eq. (1) by using *C*_*leak*_ from Eq. (2) and constant *C*_*loss*_. The analytically-calculated *C*_*leak*_ is plotted in Fig. 3(b), while the constant *C*_*loss*_ is obtained from *C*_*leak*_ for *A* = 1, i.e. *C*_*loss*_ = *C*_*leak*,*A*=1_. As shown in Fig. 3(c), the absorption bandwidth analytically calculated from Eq. (3b) with end correction ($h={l}_{n}+2.1{w}_{n}$) is well-matched with the numerical results (symbols), verifying Eq. (3b). Note that the absorption bandwidth increases up to 1.6*f*_*res*_ for ${C}_{leak}=1.5{C}_{loss}$ with a peak absorption of *A* = 0.95.

With varying the neck length (*l*_*n*_) for constant neck width (*w*_*n*_), the loss rate (*C*_*loss*_) is changed while the leakage rate (*C*_*leak*_) remains the constant. For different *l*_*n*_, the cavity size is adjusted to ensure the constant resonance frequency. As shown in Fig. 3(d), from the perfect absorption (*A* = 1; ${C}_{loss}={C}_{leak}$), the peak absorption decreases with *l*_*n*_ since *C*_*loss*_ becomes larger than *C*_*leak*_. The estimated peak absorption (dashed line) is obtained by assuming a linear increase of ${C}_{loss}\cong 0.4{l}_{n}/{l}_{n,0}$ with *l*_*n*,0_ the neck length for ${l}_{n,0}/{w}_{n}=0.5$ (Fig. 3(e)). Despite the increase in *C*_*loss*_, there is only a slight change in absorption bandwidth in Fig. 3(f) because of the increase of the vibration mass, as we have confirmed it without lossy foams. The estimated bandwidth (dashed line) is obtained by the assumed vibrating mass (Fig. 3(e)).

To demonstrate an absorption-bandwidth increase through judicious inclusion of lossy media, we fabricate different types of absorbers based on Design III: broadband absorbers (one-port system), sparse acoustic absorbers (two-port system), and duct absorbers (two-port system). We observe a significant increase in the absorption bandwidth of these absorbers.

### One-port broadband absorbers

**Figure 4:**
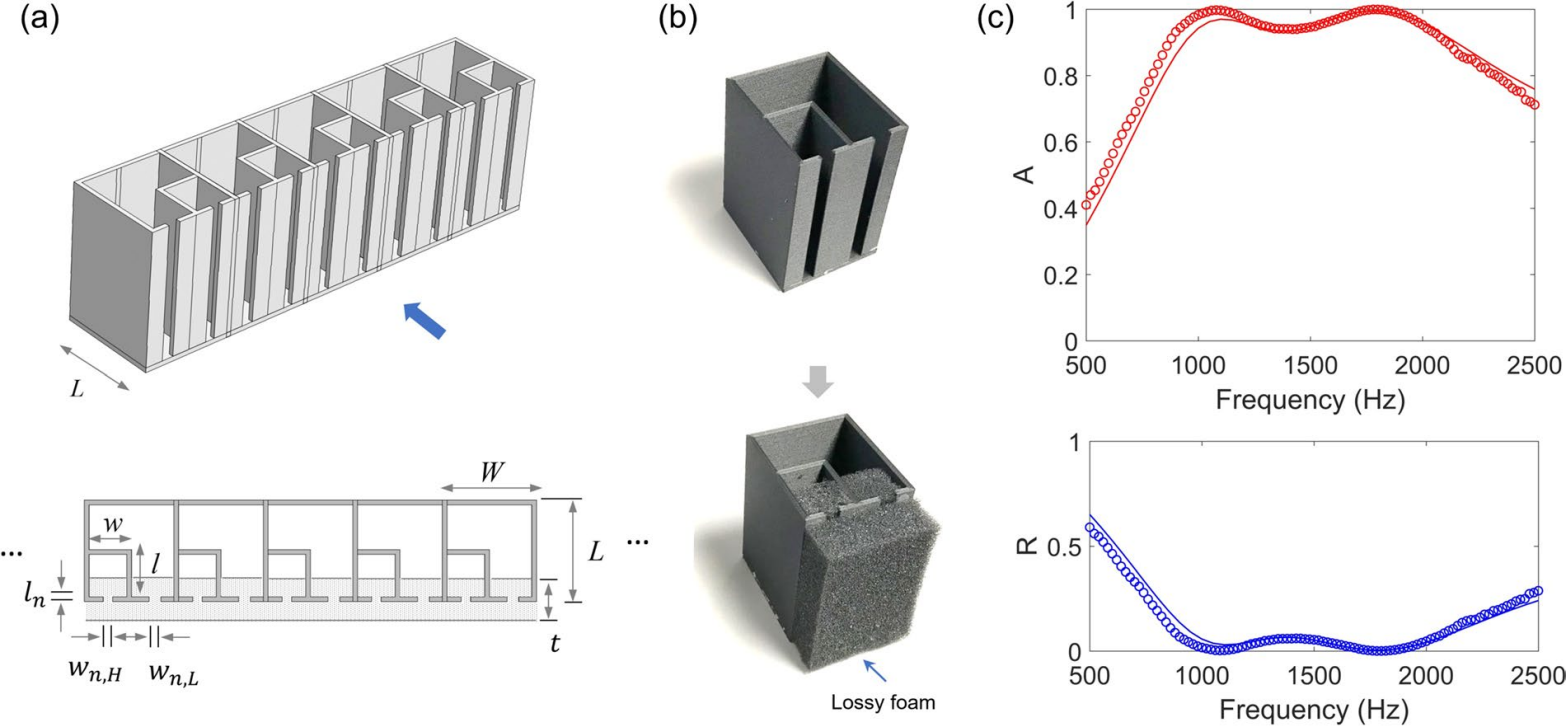
Broadband Helmholtz resonators in a one-port system. (**a**) Helmholtz resonators (period *W* and length *L*) consisting of dual-resonance resonators partially filled with foams. The two necks have similar widths (*w*_*n*, L or H_ = 5 or 4 mm), while the low-frequency resonator (*WL* − *wl*) has a larger cavity size than the high-frequency resonator (*wl*). (**b**) Prototypes of the unit device with and without foams. (**c**) Absorption (A) and reflection (R) spectra. The symbols represent the measurement data, while the solid lines indicate the numerical results.

Using damped resonances (Design III), we minimize the number of resonances in a one-port system, while obtaining broadband absorption. Figure 4(a) illustrates a one-port acoustic absorber consisting of dual resonances (without the top plate). The low-frequency resonator has a larger cavity area (*WL* − *wl*), which surrounds the smaller cavity (*wl*) of the high-frequency resonator. The two resonators have the similar neck dimensions (*w*_*n*, L or H_ = 5 or 4 mm, *l*_*n*_ = 2 mm) and are partially filled with a lossy material with a combined thickness of *t* = 20 mm + *l*_*n*_ (Design III), as the 3D-printed prototypes of the unit cell with and without the foam are shown in Fig. 4(b).

Figure 4(c) shows the acoustic absorption (*A*) and reflection (*R*) spectra of the dual-resonance absorber (*W* = 40 mm, *L* = 43 mm, *w* = 18 mm, *l* = 20 mm). We observe that high acoustic absorption (>0.8) extends from 800 Hz to 2500 Hz. Accordingly, in the high-absorption frequency band, the acoustic reflection is significantly low (bottom plot). The resonance frequencies of the two resonators are approximately 1000 Hz and 1800 Hz, respectively. The experimental results (symbols) show a good agreement with the numerical results. Note that the high-frequency resonator has a broader bandwidth than the low-frequency one (i.e. $\Delta {f}_{0.5,H} > \Delta {f}_{0.5,L}$), because the bandwidth is proportional to the resonance frequency ($\Delta {f}_{0.5}\propto {f}_{res}$ from Eq. (3a)) and the same thickness of the foam is more lossy in the high frequency (i.e. increase in *C*_*loss*_). The thickness of the foam is much smaller than the resonance wavelengths of the both resonators.

**Figure 5:**
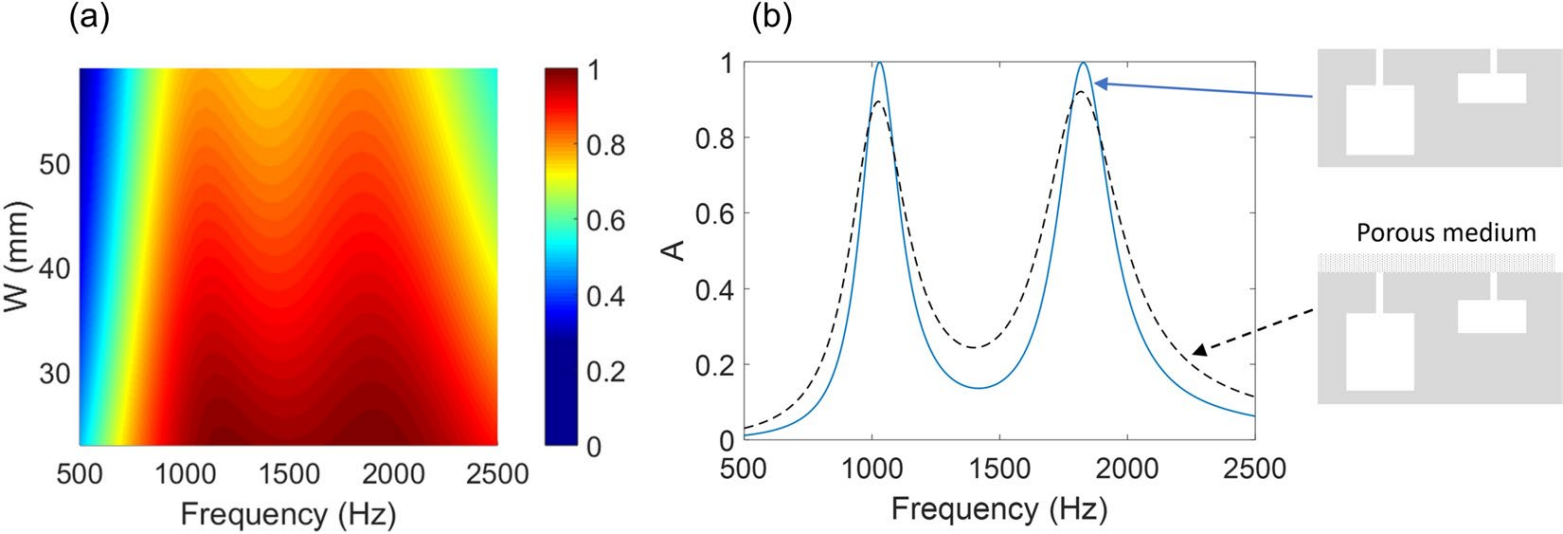
Bandwidth of the dual-resonance Helmholtz resonators. (**a**) Absorption spectra of damped resonators with respect to the period (*W*). (**b**) Absorption spectrum of the dual-resonance resonators without foams. The resonators have small neck widths (*w*_*n*, H or L_ = 0.7 mm) for the perfect absorption. The dashed line represents the same resonator overlaid with a thin foam layer (2 mm thick).

The absorption bandwidth can be increased by decreasing the period (*W*) and thus increasing *C*_*leak*_, as shown in Fig. 5(a). Increasing *C*_*leak*_ through smaller *W* is feasible as long as it does not compromise the peak absorption. The period (*W*) cannot be practically too small, because the constituent resonators need spaces occupied by their cavities; for smaller *W*, a larger *L* is needed to keep the resonance frequencies unchanged. Without the use of the foam, the absorption bandwidth is very small. Figure 5(b) shows the absorption spectrum for a dual-resonance resonator with the same period *W* = 40 mm, but without foam. We observe perfect absorption for very small neck widths (*w*_*n*, L_ = *w*_*n*, H_ = 0.7 mm). These small neck widths lead to small radiation leakage (*C*_*leak*_) that can be matched with small intrinsic losses without foams (*C*_*leak*_ = *C*_*loss*_). In addition, each resonance has a very narrow bandwidth, compared to Fig. 4(c). In Fig. 5(b), the absorption spectrum (dashed line) is plotted for the same device of the small neck with a thin foam plate (2 mm) on top, showing a decrease in peak absorption and a slight increase in absorption bandwidth because of *C*_*loss*_ > *C*_*leak*_.

### Broadband sparse absorbers

**Figure 6:**
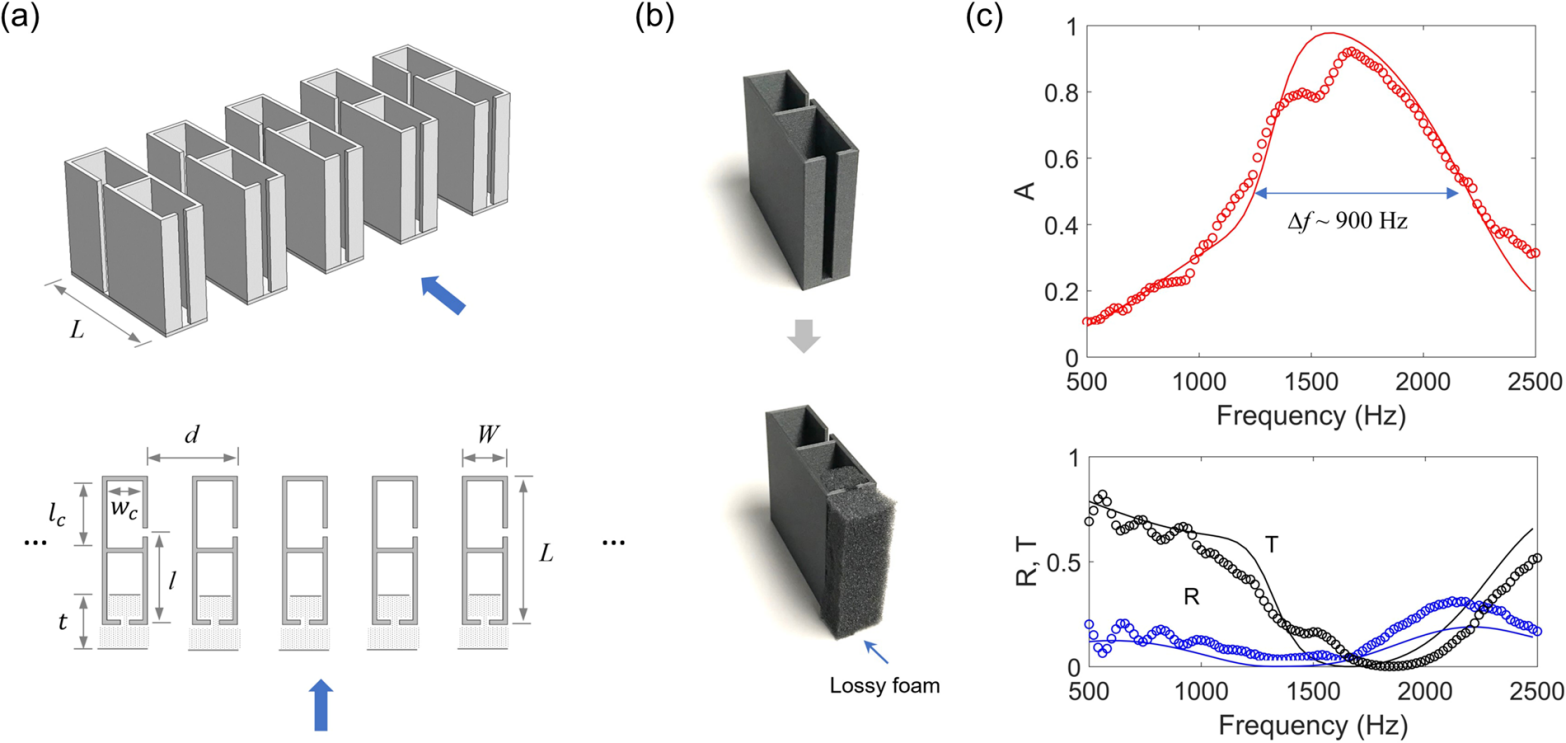
Broadband sparse absorbers. (**a**) Helmholtz resonators (period *d*, width *W* and length *L*) consisting of dual-resonance resonators: the upstream resonator partially filled with foams and the downstream resonator with no foam. The cavity sizes of the two resonators are identical (*w*_*c*_ = 16 mm, *l*_*c*_ = 30 mm), and the distance between the necks of the resonators is given by *l*. (**b**) Prototypes of the unit device with and without foams. (**c**) Absorption (*A*), transmission (*T*) and reflection (*R*) spectra. The symbols represent the measurement data, while the solid lines indicate the numerical results.

Sparse absorbers are composed of an array of resonator pairs, which are sparsely arranged, as illustrated in Fig. 6(a). Such a sparse arrangement falls into two-port systems, allowing acoustic transmission channels (Fig. 1(a)). The sparsity of the resonator is defined as the width (*W*) of the resonator to the ratio of the period (*d*) (i.e. *W*/*d*), and a high sparsity is desired in minimizing fluid flow resistance^29^. Each resonator pair consists of two resonators of the same resonance frequency, but only the resonator, facing acoustic sources, is combined with the lossy foam (Design III), as shown in Fig. 6(b). The other resonator without a lossy medium is considered a lossless resonator. The pair of the lossy/lossless resonators leads to the perfect absorption, when the distance (*l*) between the two necks of the resonators is chosen to cancel the reflected waves from the lossless resonator by the reflected waves from the lossy resonator (Fig. 1(b)). To meet the distance requirement ($l\sim \lambda /4$), the neck of the lossless resonator is positioned on the side wall.

Figure 6(c) shows the absorption (*A*), transmission (*T*), and reflection (*R*) spectra of the sparse resonators (*W* = 20 mm, *L* = 66 mm, *l* = 42 mm, *w*_*n*_ = 4 mm, and *l*_*n*_ = 2 mm) with a period of *d* = 40 mm. The cavity sizes of the two resonators are identical (*w*_*c*_ = 16 mm, *l*_*c*_ = 30 mm) for the same resonance frequency. We observe that the absorption bandwidth (Δ*f*_0.5_) is approximately 900 Hz. Notably, despite the high sparsity (*W*/*d*), the acoustic transmission is near zero around the resonance frequency. The resonance frequency is 1600 Hz. The experimental results (symbols) show a good agreement with the numerical result (solid lines).

**Figure 7:**
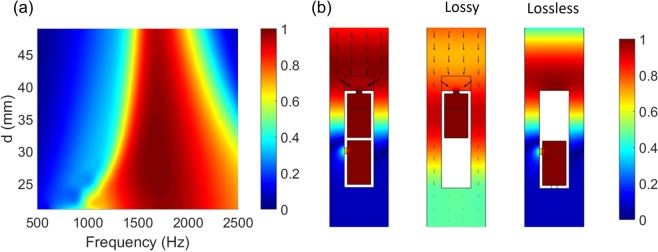
Bandwidth and pressure fields of the sparse resonators. (**a**) Absorption spectra of the sparse absorbers with respect to the period (*d*). (**b**) Pressure fields for lossy/lossless, lossy alone, and lossless alone.

Our approach for increasing the absorption bandwidth with lossy media is effective in the design of sparse absorbers that need a high sparsity. By minimizing the number of resonances, the sparse absorber is enabled with a compact structure of a high sparsity. Similar to the broad absorber in a one-port system, the absorption bandwidth increases with decreasing *d*, as shown in Fig. 7(a). Alternatively, to increase the absorption bandwidth, one can consider adding another layer of sparse resonators of a different frequency, resulting in dual resonances, unless fluid flow resistance is significantly compromised. Figure 7(b) shows pressure fields for resonators of lossy/lossless, lossy alone, and lossless alone. For the lossy/lossless case, all the acoustic energy (black arrow) goes to the front lossy resonator, whereas the lossy resonator alone permits considerable acoustic transmission. For the lossless resonator alone, acoustic wave is reflected back without transmission.

### Broadband duct absorbers

**Figure 8:**
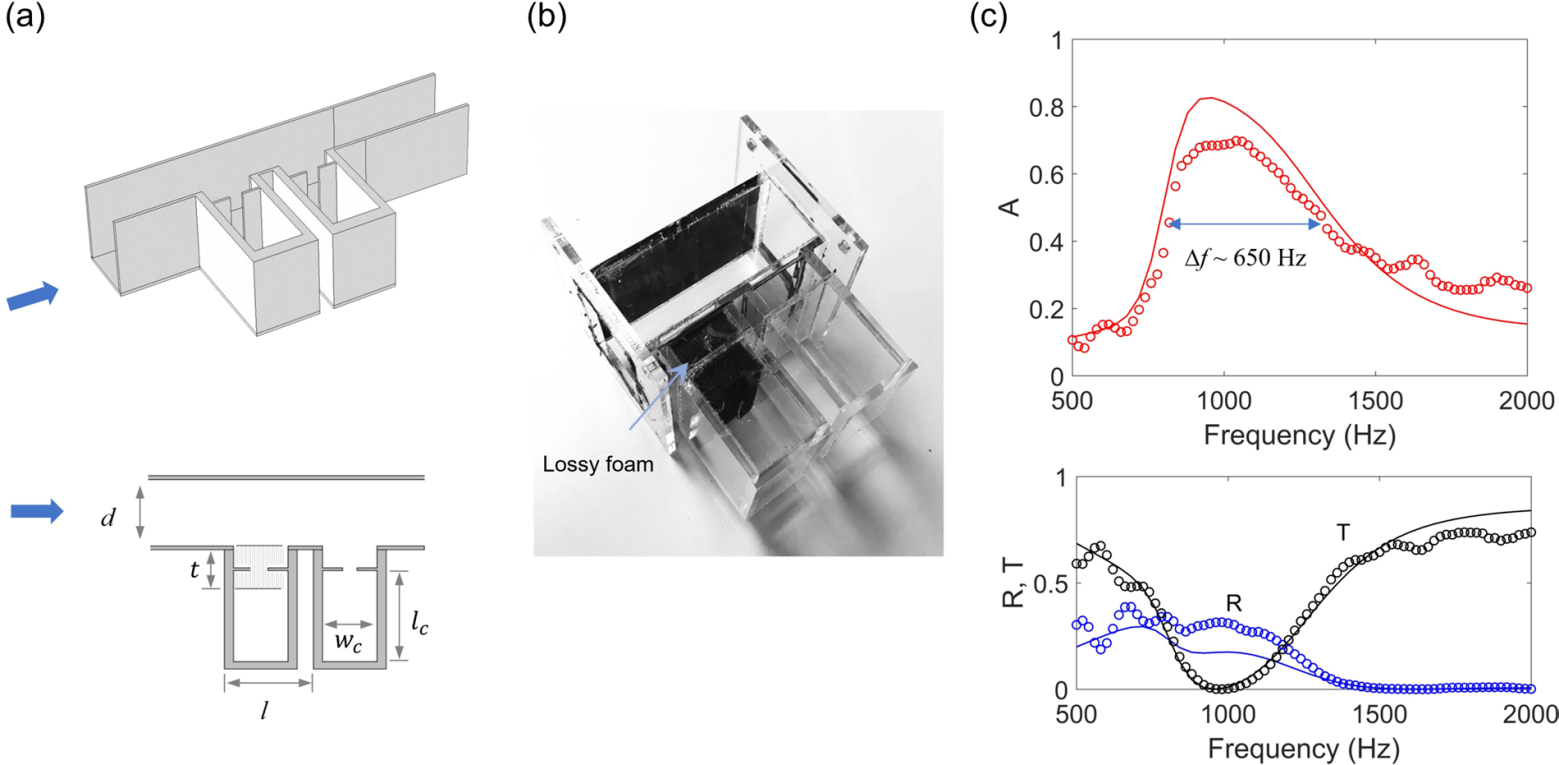
Broadband duct absorbers. (**a**) Duct absorbers (duct width *d*) consisting of identical resonators: the upstream resonator partially filled with foams (*t* = 21.5 mm) and the downstream resonator with no foam. The cavity sizes of the two resonators are identical (*w*_*c*_ = 30 mm, *l*_*c*_ = 50 mm), and the distance between the necks of the resonators is given by *l*. (**b**) Prototype of the unit device. (**c**) Absorption (*A*), transmission (*T*) and reflection (*R*) spectra. The symbols represent the measurement data, while the solid lines indicate the numerical results.

Our approach for the broadband sparse absorbers can be extended to broadband duct absorbers, as these two types of absorbers operate in two-port systems. In addition, for $d\ll \lambda$, the periodic boundary of the sparse absorber can be replaced by the hard-reflecting boundary, and thus the same physics can be applied to both the sparse absorber and duct. Broadband duct absorbers enable lots of practical applications such as HVAC (Heating, Ventilation, and Air Conditioning) systems for vehicles and buildings. Our duct absorber implementing damped Helmholtz resonators is illustrated in Fig. 8(a). Based on the perfect absorption mechanism in two-port systems (Fig. 1(b)), we use a lossy/lossless combination of resonators of the identical resonance frequency; the constituent resonators have the same size (*w*_*n*_ = 10 mm, *l*_*n*_ = 1.5 mm, *w*_*c*_ = 30 mm, and *l*_*c*_ = 50 mm). The upstream resonator only includes a lossy medium around its neck (a thickness of *t* = 21.5 mm), while the downstream resonator is considered lossless because it has a large radiation leakage and a small loss owing to its short length and large width of the neck (i.e. ${Q}_{leak}\gg {Q}_{loss}$). The distance (*l*) between these two resonators is chosen to cancel the reflected waves from the lossy resonator with the reflected waves from the lossless resonator. As shown in Fig. 8(b), the prototype of the duct absorber is fabricated with transparent acrylic plates, allowing to see the black foam in the lossy resonator.

Figure 8(c) shows the absorption (*A*), transmission (*T*), and reflection (*R*) spectra of the duct noise absorber (*l* = 49 mm, *d* = 40 mm). The numerical peak absorption is approximately 90% at the resonance frequency of *f*_*res*_ = 1000 Hz, while the measurement shows the peak absorption is about 70%. We observe the absorption bandwidth of ~650 Hz (=0.65*f*_*res*_) compared to ~0.2*f*_*res*_ without a lossy inclusion. Although the numerical results (solid lines) captures well the spectral characteristics of the experimental results (symbols), such a discrepancy between the numerical and experimental results comes unexpectedly large reflection in the measurement, possibly because of a small size difference between the two cavities and thus unwanted resonance de-tuning.

**Figure 9:**
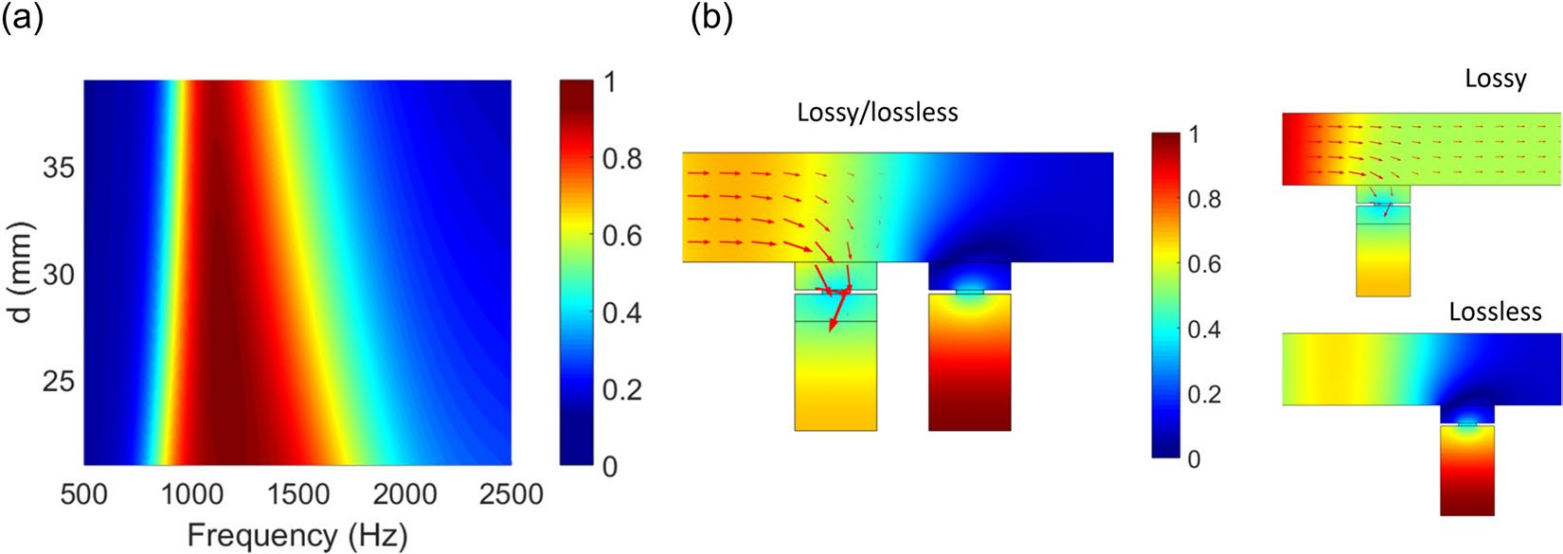
Bandwidth and pressure fields of duct absorbers. (**a**) Absorption spectra of the duct absorbers with respect to the duct width (*d*). (**b**) Pressure fields for duct absorbers composed of lossy/lossless HRs, lossy HR alone, and lossless HR alone. The arrows represent the acoustic intensity field. For lossless HR alone, there are no arrows because of perfect reflection.

The absorption bandwidth can be further increased by reducing the width (*d*) of the duct and thus increasing the radiation leakage (*C*_*leak*_), as shown in Fig. 9(a). For example, the radiation leakage is increased to *C*_*leak*_ = 1.5*C*_*loss*_ (i.e. a small deviation from the critical coupling condition), which leads to a 25% bandwidth increase (From Eq. (4a)) without significantly compromising peak absorption, i.e. $A=\tfrac{4(1.5{C}_{loss}){C}_{loss}}{{(1.5{C}_{loss}+{C}_{loss})}^{2}}=0.96$. Figure 9(b) shows pressure fields for Helmholtz resonators of lossy/lossless, lossy alone, and lossless alone. The acoustic energy is concentrated on the lossy resonator with no transmission. For lossy alone, considerable transmission is observed. For lossless alone, no transmission is seen, since the lossless resonator works as a reflector.

De-tuning approach (Fig. 2(b)) has been used for perfect duct absorbers^28,39^, but it may be ineffective in realizing broadband absorption through damped resonators. Damped resonators require a large de-tuning (${f}_{res,H}-{f}_{res,L}$) for out-of-phase responses at $f=({f}_{res,H}+{f}_{res,L})/2$. We expect that the length (*l*) of our prototype device can be further optimized to a length of $l= \sim \,0.2{\lambda }_{res}$.

## Conclusions

We have demonstrated broadband perfect acoustic absorption by using lossy porous media in combination with acoustic resonators. By placing the lossy materials around the regions of the resonators, where acoustic energy is concentrated, we have shown a significant increase in absorption bandwidths (>100% of the resonance frequency), while minimizing the use of the lossy materials. Using the damped resonances, we have demonstrated bandwidth increases for three types of acoustic absorbers. Our design approach can benefit acoustic absorbers based on Helmholtz resonators for broadening absorption bandwidths.

## Methods

### Sample fabrication

The two types of absorbers (one-port broadband absorbers and sparse absorbers) are fabricated by using a fused-deposition-molding (FDM) style 3D printer (Model: Markforged Mark Two, Markforged Inc., MA, USA) with 400 µm nozzle diameter and 100 µm layer height. The duct absorbers are made of acrylic plates with a thickness of 5 mm. The 3D-printed and acrylic resonators are partially filled with lossy foams (polyurethane foam, Monoprice Inc., CA, USA).

### Experiments setup

Acoustic performance is characterized with an in-house impedance tube measurement system, consisting of an impedance tube with a square cross section (40 × 40 mm^2^), a full-range speaker (2½”, model: SB65WVAC25-4, SB Acoustics, http://www.sbacoustics.com/), pressure-field microphone and preamplifier (¼” prepolarized, sensitivity: 1 mV/Pa, model: 378C10, PCB Piezotronics, NY, USA), audio power amplifier (model: APA150, Dayton Audio, OH, USA), data acquisition device (24-bit, 102.4 kS/s, model: NI USB-4431, National Instruments). For characterization of two-port systems, the four-microphone measurement method is employed with pyramid-shaped anechoic termination (polyurethane foam), while the two-microphone measurement method is used for one-port systems. The measured signals are processed with Matlab data acquisition toolbox (Mathworks, MA, USA).

### Numerical calculation

Acoustic pressure fields are calculated by a commercial finite-element method solver, COMSOL Multiphysics 5.3. To simulate acoustic absorption in porous layers, empirical models of the complex acoustic impedance (*Z*_*f*_) and complex wave number (*k*_*f*_) are used^40^, which are given, respectively, by

4 $${Z}_{f}=\rho c[1+{c}_{1}\,{f}^{{c}_{2}}+i{c}_{3}\,{f}^{{c}_{4}}],$$

5 $${k}_{f}=\frac{2\pi f}{c}[1+{c}_{5}\,{f}^{{c}_{6}}+i{c}_{7}{f}^{{c}_{8}}],$$

where *c*_1_ − *c*_8_ are the constants (*c*_1_ = 74891, *c*_2_ = −1.8432, *c*_3_ = −1489000, *c*_4_ = −2.404, *c*_5_ = 42, *c*_6_ = −1.0813, *c*_7_ = −11.5412, and *c*_4_ = −0.4794) extracted by acoustic measurement (see Supplementary Materials). In COMSOL, with Eqs (4) and (5), the material properties of the foam are defined by the complex sound speed (${c}_{f}=2\pi f/{k}_{f}$) and complex mass density (${\rho }_{f}={Z}_{f}/{c}_{f}$).

### Harmonic oscillator model

The equation of motion of the one-dimensional (1D) harmonic oscillator is described by

6 $$\frac{{d}^{2}x(t)}{d{t}^{2}}+\frac{({C}_{loss}+{C}_{leak})}{{m}_{0}}\frac{dx(t)}{dt}+{\omega }_{0}^{2}x(t)=\frac{F(t)}{{m}_{0}},$$

where *x*(*t*) is the oscillating amplitude, and *F*(*t*) is the force acting on the oscillator. In the regime of the period *d* that is much smaller than the wavelength *λ*, we have the forms of $F(t)=2p(t){S}_{osc}$ and ${\eta }_{r}={Z}_{s}{S}_{osc}^{2}/{S}_{inc}$, respectively, where *p*(*t*) is the pressure of the incident acoustic wave, *Z* is the acoustic impedance of the surrounding fluid, *S*_*osc*_ is the area of the oscillator, and *S*_*inc*_ is the area of the unit cell. By solving Eq. (6), the oscillating amplitude in the frequency domain (*ω*) is given by

7 $$x(\omega )=\frac{F(\omega )}{{m}_{0}}\frac{1}{({\omega }_{0}^{2}-{\omega }^{2})-i(\frac{{C}_{loss}}{{m}_{0}}+\frac{{C}_{leak}}{{m}_{0}})\omega }.$$

The dissipation power of the oscillator is represented by

8 $$\begin{array}{rcl}{P}_{diss} & = & \frac{1}{2}\frac{{C}_{loss}}{{C}_{loss}+{C}_{leak}}Re[{F}^{\ast }(t)\frac{dx(t)}{dt}]\\  & = & \frac{8\frac{{C}_{loss}}{{C}_{c}}\frac{{|p(\omega )|}^{2}{S}_{osc}^{2}}{{C}_{0}}{\omega }_{0}^{2}{\omega }^{2}}{{({\omega }_{0}^{2}-{\omega }^{2})}^{2}+4{(\frac{{C}_{loss}}{{C}_{c}}+\frac{{C}_{leak}}{{C}_{c}})}^{2}{\omega }_{0}^{2}{\omega }^{2}}.\end{array}$$

By using Eq. (8) and the incident power ${P}_{inc}=\frac{1}{2}\frac{|p(t){|}^{2}{S}_{inc}}{{Z}_{s}}$, the acoustic absorption (power) coefficient, the ratio *P*_*diss*_/*P*_*inc*_, is expressed by

9 $$A(\omega )=\frac{16(\frac{{C}_{leak}}{{C}_{c}}\frac{{C}_{loss}}{{C}_{c}})}{{(\omega /{\omega }_{0}-{\omega }_{0}/\omega )}^{2}+4{(\frac{{C}_{leak}}{{C}_{c}}+\frac{{C}_{loss}}{{C}_{c}})}^{2}}.$$

The peak absorption at resonance ($\omega ={\omega }_{0}$) is given by

10 $$A=\frac{4{C}_{leak}{C}_{loss}}{{({C}_{leak}+{C}_{loss})}^{2}}.$$

Perfection absorption occurs when *C*_*leak*_ = *C*_*loss*_, i.e. critical coupling condition. Rigorously, the critical coupling condition states that the radiated power (*P*_*leak*_) is balanced with the dissipated power (*P*_*loss*_):

11 $${P}_{leak}={P}_{loss}.$$

These power forms are given by ${P}_{leak}=\frac{1}{2}{C}_{leak}{|\frac{dx(t)}{dt}|}^{2}$, and ${P}_{loss}=\frac{1}{2}{C}_{loss}{|\frac{dx(t)}{dt}|}^{2}$, where $\frac{dx(t)}{dt}$ is the root-mean-square average of vibration velocity. Thus, the equality of *P*_*leak*_ = *P*_*loss*_ implies *C*_*leak*_ = *C*_*loss*_.
